# Assessing the sustainability of freshwater systems: A critical review of composite indicators

**DOI:** 10.1007/s13280-016-0792-7

**Published:** 2016-06-01

**Authors:** Derek Vollmer, Helen M. Regan, Sandy J. Andelman

**Affiliations:** 1 Betty and Gordon Moore Center for Science, Conservation International, 2011 Crystal Drive, Suite 500, Arlington, VA 22202 USA; 2 Department of Biology, University of California, Riverside, CA 92521 USA

**Keywords:** Freshwater, Indicators, Index, Social-ecological system, Sustainability assessment

## Abstract

Quantitative indicators are a common means of assessing the complex dimensions of a sustainable freshwater system, and framing scientific knowledge for policy and decision makers. There is an abundance of indicators in use, but considerable variation in terms of what is being measured and how indicators are applied, making it difficult for end-users to identify suitable assessment methods. We review 95 water-related indices and analyze them along their normative, procedural, and systemic dimensions to better understand how problems are being defined, highlight overlaps and differences, and identify the context(s) in which a particular index is useful. We also analyze the intended use, end-users, and geographic scale of application for each index. We find that risk assessment is the most common application (*n* = 25), with indices in this group typically focusing either on hazard identification (biophysical assessments) or vulnerability of human populations. Indices that measure freshwater ecological health are not explicitly linking these indicators to ecosystem services, and in fact the concept of ecosystem services is rarely (*n* = 3) used for indicator selection. Resource managers are the most common group of intended end-users (*n* = 25), but while 28 indices involved consultation with potential end-users, 11 did not specify an intended use. We conclude that indices can be applied as solution-oriented tools, evaluating scenarios and identifying tradeoffs among services and beneficiaries, rather than only assessing and monitoring existing conditions. Finally, earlier engagement of end-users is recommended to help researchers find the right balance among indices’ salience, legitimacy, and credibility and thus improve their decision relevance.

**Electronic supplementary material:**

The online version of this article (doi:10.1007/s13280-016-0792-7) contains supplementary material, which is available to authorized users.

## Introduction

Freshwater systems sustain people, underpinning agricultural production, industrial processes, urban development, and other biota that we depend on, and yet we are consistently reminded that these systems are in crisis. Recent global analyses have highlighted the link between water scarcity, food insecurity (Rockström et al. 2009a; Brauman et al. 2013), and poverty (WWAP 2015), and have documented trends in freshwater ecosystem degradation (Gardner et al. 2015), aquifer depletion (Richey et al. 2015), and declines in aquatic biodiversity (Strayer and Dudgeon 2010; Dudgeon 2014). Climate change and ongoing human population growth are expected to accelerate many of these negative trends (Vörösmarty et al. 2000; Alcamo et al. 2007; Palmer et al. 2008; Padowski and Gorelick 2014). The impending global water crisis was underscored more than two decades ago (Gleick 1993) and its importance and imminence have amplified among researchers (Vörösmarty et al. 2010; Srinivasan et al. 2012; Green et al. 2015) and decision makers (2030 Water Resources Group 2009; Cooley et al. 2014) recently. In the World Economic Forum’s (2015) Global Risks report for 2015, survey respondents ranked water crises the number one risk in terms of societal impact, above infectious diseases, weapons of mass destruction, and fiscal crises. While much progress has been made in identifying the complex problems related to the sustainability of freshwater systems, there has been less success in identifying solutions (Pahl-Wostl et al. 2013), the typical gap between what science offers and what decision makers need.

One area where science and policy converge around water resources is in the field of indicators, the “component[s] or measure[s] of environmentally relevant phenomena used to depict or evaluate environmental conditions or changes or to set environmental goals” (Heink and Kowarik 2010). In principle, indicators should be sensitive to changes over time, refer to benchmark or threshold values, be predictive or anticipatory and convey relevance to the stated objectives of assessment (Liverman et al. 1988). Indices are increasingly employed to benchmark performance on a range of issues and, if constructed well, can offer a powerful communication and management tool (OECD 2008), but are also prone to oversimplifying complex issues of sustainable development and not adequately reflecting the state of the science (Böhringer and Jochem 2007; Niemeijer and de Groot 2008). Although indicators are typically designed to meet the informational needs of policy and decision makers (Lorenz et al. 2001), they are often dependent on, and derived from, scientific knowledge and methods (Turnhout et al. 2007).

Indicators have long been used to help calculate risks, monitor changes, measure progress and, more recently, plan for greater sustainable use of water resources. Physical and chemical water quality metrics have been in use since the 1960s; in the 1980s, biological metrics were introduced as an alternative way to assess freshwater ecological integrity, and by the 1990s these and other metrics of ecological stress were routinely used to reflect the state of the environment (Spangenberg and Bonniot 1998). Seminal work on human dependence on water resources (Falkenmark et al. 1989; Gleick 1996) helped spur further research into quantifying normative concepts such as water stress, vulnerability, and sustainability.

These definitions matter—each has theoretical underpinnings as well as value judgments that, in turn, suggest different methods with different implications for policy and prioritization (Laderchi et al. 2003). Water stress, for example, can refer to physical availability for food production, or the delivery of water resources for direct human consumption (sometimes referred to as “economic” scarcity) (Rijsberman 2006). Quantifying stress then requires decisions on what constitutes a water source (e.g., soil moisture) (Rockström et al. 2009a), whether and how water is shared between human and ecological needs (Smakhtin et al. 2004), and whether local infrastructure exists to deliver and clean this water (McDonald et al. 2014). Over time, researchers and practitioners have developed composite indicators (hereinafter referred to as “indices”) to capture more complex dynamics between the social and ecological components of water resource supply and demand, meet more specific informational needs, utilize new forms of data, and integrate knowledge from multiple disciplines. This proliferation has created a surfeit of information related to the sustainability of freshwater systems, making it difficult for end-users to navigate let alone understand and identify the most appropriate assessment methods for their informational needs.

Here, we offer a review of existing water-related indices, elucidating the various dimensions from problem definition to indicator aggregation, with the aim of providing insights that can be useful to the research, policy, and practitioner communities interested in identifying appropriate methods for assessing the sustainability of freshwater systems. We systematically review 95 indices using Binder et al.’s (2010) framework for evaluating sustainability assessment methods. We apply this framework to identify the different analytical lenses authors have applied, which we consider a combination of Binder et al.’s (2010) normative dimension (problem definition, goal-setting, and assessment type) and systemic dimension (parsimonious yet sufficient representation of the main structures, processes, and functions of the system being studied). Previous reviews have addressed indices applying a single analytical lens, such as risk assessment (Brown and Matlock 2011; Plummer et al. 2012; Doczi 2014; Pedro-Monzonís et al. 2015) or life-cycle analysis (Kounina et al. 2012), but we provide the first comprehensive review that compares these different approaches and their relative merits and limitations in the context of supporting decision makers. Next, we evaluate the procedural dimension—the likely end-users, involvement of stakeholders in index development, as well as the geographic scale at which these indices can be applied. Finally, we conclude by discussing research gaps and potential opportunities for further improvements to water-related indices.

## Methodology

We examined both the peer-reviewed and gray literature, since some indices are the product of managerial needs rather than academic research. We established three simple criteria for selection of indices to include in the review. First, we only considered indices for which water is the focus (including water supply, disaster risk reduction, and ecological assessments), rather than a sub-component or sub-index of a larger composite such as the Environmental Sustainability Index (Esty et al. 2005). This was an attempt to capture the broad range of human–water interactions, while maintaining a reasonable scope. Second, we evaluated indices rather than individual indicators (e.g., phosphorous concentrations) because the former represents a deliberate attempt to select the most appropriate indicators and combine them to provide a concise yet comprehensive assessment of freshwater systems. However, it should be noted that the distinction is not always clear, as indicators themselves can be a composite of more than one metric. Moreover, not all indices are aggregated into a final value or classification, thus many indices evaluated in this review are in fact an assemblage of indicators. Finally, we only reviewed indices with documented and transparent methods. Proprietary assessment methods such as those used by consultancies to produce risk assessments were excluded, as were indices that do not provide enough information to reproduce them. Application of these criteria resulted in selection of 95 indices for review (see Table S1 Supplementary Material for the full list of references).

In addition to reviewing the 10 indices cited within existing review papers (Brown and Matlock 2011; Juwana et al. 2012; Plummer et al. 2012; Hester and Little 2013; Doczi 2014; Pedro-Monzonís et al. 2015), we conducted online searches using Web of Science and Google, the latter in an attempt to capture publicly available gray literature, using six different search terms:

- Water sustainability index,
- Water health index,
- Water risk index,
- Water vulnerability index,
- Water quality index, and
- Water sustainability indicators.

For Google searches, we viewed the first 200 records returned. For Web of Science searches, we did not specify a date range but restricted records to those with at least one citation. We only conducted searches using English and thus our results are constrained to the English language literature. We excluded papers that merely implemented a previously developed index, unless the authors claimed to have made modifications sufficient to qualify it as a distinct index, e.g., Perez-Foguet and Garriga’s (2011) Enhanced Water Poverty Index (eWPI) builds on Sullivan et al.’s Water Poverty Index (2003) and hence was included in our review as a separate index.

In an effort to categorize the indices and discern the reasons for the apparent diversity of approaches (Plummer et al. 2012), we evaluated each index against several criteria: the analytical lens that the authors employed to select indicators; their intended end-user and primary or expected use; the spatial scale of application and any spatial differentiation used to compare sub-units; category (or type) and number of indicators; and the type of aggregation rules (if any) applied to arrive at a final index score (Table 1). Most of these criteria are clearly addressed within each paper, although the first two criteria are open to ambiguity and, if not stated directly within the documentation, required a judgment on their classification.

**Table 1:** Specific criteria used to evaluate selected indices

Criteria	Subcriteria
Analytical lens	How is the problem or issue defined?
	How is the system represented? What components are being measured?
End use	What type of decision contexts is the index designed for? Can it be used dynamically?
	How, if at all, have end-users been involved in the development?
Data representation	What, if any, weighting and numeric aggregation methods are used?
	What is the spatial unit of analysis?
	What are the data collection requirements?

Indices vary considerably in terms of their demonstrated applications, something that is not easily discerned through research citations or website visits and so we do not attempt to evaluate the effectiveness of any particular index. There is also substantial variation in the amount of documentation available, and the resources that have been invested in their development. Thus, our aim was not to compare indices against one another, but to examine the breadth of indices, orient potential users to the variety of approaches, and highlight potential gaps and opportunities for further development of indices for decision support.

### Identifying analytical lenses

In principle, indices ought to be based on a conceptual framework which defines the phenomenon and goals of interest, identifies important sub-components, and guides the selection of indicators and data needs (Walmsley 2002; OECD 2008). Yet many indices are criticized for a lack of formal selection criteria (Dale and Beyeler 2001; Niemeijer and de Groot 2008) or rigorous conceptual grounds on which assessment goals are based (Vugteveen et al. 2006). Indicator selection reflects personal biases, technical considerations, knowledge constraints (Boulton 1999) and goals and is thus unavoidably normative (Turnhout et al. 2007; Ioris et al. 2008). Beginning with a widely accepted concept of sustainable development, authors must identify specific sectoral problems and goals that might be addressed by an assessment (Binder et al. 2010). For example, will socioeconomic indicators be included? Is sustainability understood to be intergenerational?

We use the term “analytical lens” here to denote the conceptual foundation on which indicator selection is based; many authors of the indices reviewed here refer to having created their own unique framework for indicator selection. Not all of the indices we reviewed make explicit reference to an analytical lens, but all allude to some form of logic that guides indicator selection, which we have defined and summarized in Table 2. The breadth of categories was developed iteratively, and we determined that it is not possible to delineate mutually exclusive, collectively exhaustive categories for these lenses—there are inevitable overlaps in terms of technical concepts as well as appeals to general principles of sustainability (Ferguson et al. 2012). Our aim is thus to offer an overview of where indices tend to fall, and the implications of applying a particular lens when assessing a freshwater system. Certain lenses are well established within the literature and suggest specific technical assessment methods, such as the Driving Forces-Pressures-State-Impact Response (DPSIR), ecological health, ecosystem services, life-cycle assessment, and risk assessment categories. To these we have suggested three additional analytical lenses that are distinct in terms of their scope and/or definition of the problem(s) related to sustaining freshwater systems. Some of these lenses focus on a specific, arguably under-researched element of freshwater system sustainability (e.g., infrastructure service delivery, institutional performance) and so could conceivably be nested within other, more holistic lenses.

**Table 2:** Analytical lenses used to conceptualize problems and select water-related indicators

Analytical lens	Description
DPSIR (*n* = 14)	The Driving Forces-Pressures-State-Impact-Response (DPSIR) lens is an elaboration on the Pressures-State-Response (PSR) framework developed by the Organisation for Economic Co-operation and Development (OECD) as a way to organize indicators measuring sustainability. It introduces a cause-effect logic that links human activities (pressures) to environmental impacts (state) that lead to policy and management responses
Ecological health (*n* = 15)	Ecological health uses a variety of biological, physical, morphological, and chemical proxies to compare a freshwater ecosystem to 1) a historical (or pristine) reference point (Karr, 1981) or 2) a threshold based on an ecosystem’s ability to sustain its supply of goods and services (Karr 1999). The latter is more commonly used (Boulton 1999; Karr 1996; Vugteveen et al. 2006; Korbel and Hose 2011) and is reflected by a preference for the term “health” over the previously used term “integrity”
Ecosystem services (*n* = 3)	Several frameworks exist to quantify ecosystem services—the benefits that humans obtain from nature. Evaluations generally rely on spatially explicit analysis (mapping and modeling service production [supply] and beneficiaries [demand]). Ecosystem services may be reduced to a single monetary indicator using a variety of economic valuation methods, or they may be represented in biophysical units (e.g., tons of sediment retained per year) or simply ranked (e.g., low to high value)
Infrastructure service delivery (*n* = 6)	Infrastructure service delivery is focused primarily on the water, sanitation and hygiene (WASH) sector. Indices in this category are united by their attention to issues such as the financial sustainability of infrastructure systems and the equitable distribution of and access to services, a deliberate attempt to expand indicators in this field beyond one-off assessments of infrastructure functionality (Lockwood and Le Gouais 2014)
Institutional performance (*n* = 6)	Institutional performance has recently been singled out as warranting more attention in the context of water resource management (Hooper 2010; Plummer et al. 2012). Indices in this category are means-oriented (i.e., prescribing practices thought to be more “sustainable”), primarily qualitative, such as ratings for mechanisms for coordinated decision making
Life-cycle assessment (*n* = 11)	Life-cycle assessment methods rely on inventories of material inputs and outputs for a particular process or product, to quantify its environmental impacts, reducing a substantial amount of data into a final indicator, a volume of water which constitutes the “water footprint.” This approach has recently been adapted to evaluate impacts of freshwater consumption (2005). Impacts measured include water withdrawals, consumption, and pollution, each of which can be scaled up or down
Risk assessment (*n* = 25)	Risk assessments typically consist of two major steps: (1) identifying potential water-related threats (e.g., natural hazards, physical scarcity, pollution) to human populations (Vörösmarty et al. Kounina et al. 2012); and (2) characterizing the population’s susceptibility to harm, based on its likelihood of exposure to hazards, the severity of potential outcomes, and its capacity to adapt (Adger 2006)
System sustainability (*n* = 15)	This lens emphasizes human dependence on water resources, the linkages among social, economic, and environmental sub-systems (i.e., social-ecological or coupled human-natural systems), and the intergenerational aspect of sustainability. One of the more common ways to operationalize the concept of sustainability is to separate it into dimensions, e.g., the “triple bottom line” of economic, environmental, and social indicators (Spangenberg and Bonniot 1998), while other authors define properties of a sustainable *system* and focus more attention to the interlinkages among these dimensions to (Loucks 1997)

## Results

A summarized assessment of all 95 indices, including the analytical lens, primary use, end-user(s), and geographic scale of application, appears in Table S2. The Supplementary Material also includes information on the component categories, number of indicators, and weighting procedure applied. In the following sub-sections, we elaborate on the results summarized in these tables.

### Analytical lenses

#### DPSIR

Fourteen indices refer specifically to the DPSIR or PSR lens. Liaw et al. (2000) and Walmsley (2002) were among the first authors to recommend applying the DPSIR lens to integrated catchment management and it continues to be used, most recently in global assessments (UNESCO-IHP et al. 2012; UNEP and UNEP-DHI 2015) Indices in this category generally prioritize available data, making them easy to calculate. It is often applied to individual indicators (Niemeijer and de Groot 2008), leading to three or more parameters for each indicator, e.g., Pressure, State, and Response parameters for the Policy indicator in the Watershed Sustainability Index (Chaves and Alipaz 2007). This analytical lens is widely applied in environmental management and attempts to identify causal relations, though it has been criticized for assuming linearity in these relationships (Perez-Foguet and Garriga 2011), having ambiguous categories and not accounting for ecosystem services (Kelble et al. 2013), and focusing on “end-of-pipe” remedial solutions (Spangenberg and Bonniot 1998).

#### Ecological health

Fifteen indices apply an Ecological Health lens, to a variety of aquatic ecosystems including rivers, riparian zones, lakes, wetlands, and estuaries. These indices offer the most scientifically comprehensive assessment of the state of aquatic ecosystems. Health, rather than integrity, is now the preferred nomenclature among indices in this category, reflecting the distinction many authors have made (Boulton 1999; Karr 1999; Vugteveen et al. 2006; Korbel and Hose 2011) that claim the former term is more relevant to societal values; “ecological health” allows for discussion of thresholds which provide acceptable or desirable levels of ecosystem services while maintaining ecological function (Karr 1996). Many of these indices make reference to ecosystem services as the endpoints humans value but stop short of quantifying the services or the relationship between ecological health and service provision, nor do they set the thresholds for “healthy” freshwater systems, i.e., connecting quantitative values of the index to what might be acceptable levels of ecosystem health (Beck and Hatch 2009). Populating these indices usually requires direct examination of affected biota, and reference points are determined regionally, but this also limits their application in areas where data and resources are sparse, and where users seek to make explicit connections to human well-being.

#### Ecosystem services

Despite its emphasis on quantification and connections to human well-being (Summers et al. 2012), only three of the indices we reviewed explicitly rely on an ecosystem services analytical lens to define indicators (Abel et al. 2003; Tipa and Teirney 2006; Dodds et al. 2013), while two others included a number of indicators that were classified as ecosystem services (Smajgl et al. 2010; van Leeuwen et al. 2012). Even among these, there is substantial variation among the services measured: Abel et al. (2003) involved stakeholders directly in the identification and ranking of ecosystem services in an Australian catchment; Dodds et al. (2013) include disturbance and water quality regulation indicators but rely exclusively on biophysical indicators and global datasets, Tipa and Teirney (2006) focus on spiritual and symbolic cultural services, and van Leeuwen et al. (2012) include biodiversity and esthetic cultural services. These disparities highlight some challenges to applying an ecosystem services lens to freshwater assessments. Beyond water provisioning (which most indices address, regardless of their analytic foundation), there is considerable debate as to the appropriate measures of other water-related services (Vollmer et al. 2016) and whether these services should be further reduced to monetary indicators (Kallis et al. 2013) as is frequently done. This also means that our review inadvertently excluded ecosystem service assessments that did not explicitly refer to indicators.

#### Infrastructure service delivery

As noted in the introduction, water stress can be a result of populations affected by poor service delivery (cost and/or quality) rather than a physical scarcity of water. Infrastructure service delivery indices focus almost exclusively on water and sanitation services and the technologies that provide them, although the indicators attempt to measure the non-technical factors that affect the long-term viability of technical interventions. Five of the six indices in this group focused on WASH issues, while Bos (1997) concentrates on irrigation and drainage systems. The indices have unique emphases, such as “performance assessment” (Bos 1997), “demand-responsiveness” (Sara and Katz 2005), or reducing inequalities (JMP 2015) that appear to reflect prevailing priorities of funding agencies. Consequently, measuring these indicators requires detailed (often qualitative) field surveys.

#### Institutional performance

A small group of indices (*n* = 6) have been recently developed to track the performance of institutions (or water resource governance), often specifically to measure progress toward implementing principles of integrated water resource management (IWRM) or, as Sullivan (2010) puts it, shift more attention from hydrologic regimes to governance issues. The concept of IWRM is subject to interpretation (Cook and Spray 2012) but the general tenets of coordinating management of water and terrestrial ecosystems at a basin scale are widely accepted, and indices in this category generally draw from the expansive list of indicators proposed in Hooper (2010). These include categories such as coordinated decision making, goal completion, financial sustainability, training, and capacity building. Indices under this analytical lens are means-oriented (i.e., prescriptive in terms of what constitutes ‘sustainable practices’) (Binder et al. 2010), primarily qualitative, and are often connected to the development of River (Lake) Basin Organizations. As such, they require surveys and interviews to collect primary data for populating the indicators.

#### Life-cycle assessment (LCA)

Indices in this category (*n* = 11) span spatial scales, since they link to global datasets on water consumption, which can then be traced back to final demand in a specific locality. Footprints can be assessed at the community or basin scale by summing the water footprints in a geographic area—five of the indices were designed to be applied specifically to cities and urban water management issues. This footprint may be sub-divided into blue water (fresh surface and groundwater consumed in a process), green water (precipitation that transpires through plants without recharging aquifers), and gray water (water used to assimilate pollutant loads) (Hoekstra et al. 2009) or it may be weighted according to the quality of the water used (Bayart et al. 2014) and/or the level of water stress present in the source region. Decisions on how to normalize, weight, and aggregate data are left to expert analysts [Bohringer et al. 2007]. Large uncertainties in LCA studies suggest that their value is in signaling where there is a need for more detailed assessments of water resource consumption (Pfister et al. 2009), while some researchers question whether water footprinting should be used in policy discussions at all because it lacks sufficient information on the actual impacts of “virtual water consumption” (Wichelns 2015). That said, it may be the most suitable method for corporate decision makers seeking to manage their industries’ impacts and dependence on freshwater resources.

#### Risk assessment

Collectively, risk assessment appears to be the most common analytical lens used in the indices we reviewed (*n* = 25). These indices focus almost exclusively on water supply risks for human populations (including domestic, industrial, and agricultural water supplies), although five indices include an indicator related to flooding risk. Scarcity is generally measured using global proxies, such as Falkenmark’s indicator of water stress (1700 m^3^ renewable water resources per capita per year) or environmental water requirements (Smakhtin et al. 2004). Only four indices (Sullivan and Meigh 2005; Kang and Lee 2011; Chang et al. 2013; Devineni et al. 2013) used quantitative probabilities to characterize risks; the majority used proxies (such as counting flood occurrences over a period of time).

Based on our judgment, 12 focus primarily on hazard identification (where exposure is assumed but not measured), while 13 focus on vulnerability (where hazards may be measured but in some cases are merely assumed). The reason for this segmentation likely has to do with the data and methods available. Hazard identification mostly relies on geospatial predictors and can be constructed using hydrologic models and widely available datasets (Srinivasan et al. 2012). Vörösmarty et al. (2010) attempt to incorporate one element of vulnerability assessment (technological investments like reservoirs) into their analysis of “incident threats” to water security, and Green et al. (2015) recently extended Vörösmarty et al.’s method by rescaling the threats according to the number of people living downstream from freshwater provisioning areas as a measure of potential exposure. The Water Poverty Index (WPI) (Sullivan et al. 2003) represents a better integration of hazard identification and vulnerability assessment to characterize risk. The WPI accounts for availability of water resources, access for human use, and capacity to manage water, and later variants like the Climate Vulnerability Index (Sullivan and Meigh 2005) and Water Vulnerability Index (Sullivan 2010) both build on the foundations of the WPI.

#### System sustainability

The concept of sustainability has different interpretations across the range of indices that use the concept (*n* = 14), further illustrating the normative aspect of problem definition and indicator selection. Several of the indices in this subset refer to a “balanced picture” of sustainability, that is, an equal number of economic, social, and environmental indicators (Ioris et al. 2008). Marques et al. (2015) criticize this “triple bottom line” approach for overlooking issues such as governance and technologies. Other authors derived independent definitions for water-related sustainability, such as Schneider et al.’s (2014): contribution to societal goals of regional development, maintenance of ecological and hydrological integrity, contribution to social justice, and adaptive capacity. Despite attempts to provide a holistic picture of sustainability, some indices in this category tend to offer weak conceptual foundations, lacking the integrative element that links indicators (Singh et al. 2009). A subset within this category gives comparatively more consideration to how social and ecological indicators interrelate and, consequently, suggest more complex methods and data requirements. Loucks’s (1997) Sustainability Index formulates a framework of three indicators (reliability, resilience, and vulnerability) for measuring the sustainability of a water resource system, but only one other index (Sandoval-Solis et al. 2011) in this category refers to this framework. Four other indices (Cai et al. 2002; Bagheri et al. 2006; SWRR 2008; Shilling 2013) refer to system sustainability but present their own categories of indicators of a sustainable system, including equity and meeting consumer demand.

### Uses, end-users, and spatial scale of application

In principle, any index could be employed for multiple uses and many of the indices we reviewed allude to multiple uses. We summarize these uses in Table 3 into benchmarking and monitoring, facilitating IWRM, prioritizing investment, and raising public awareness. Indices are useful tools to measure and then communicate the current state of a freshwater system—58 of the indices we reviewed were developed primarily to provide a benchmark or to facilitate public awareness, and most (*n* = 56) of the indices are aggregated into a single index score. The decision on whether or not to aggregate is influenced by the index’s end use—a single summary statistic facilitates easy comparison and prioritization across assessment units, captures the attention of media and policy makers, and can be unpacked to reveal the component indicator values (OECD 2008). However, the aggregation process requires a decision on whether and how to weight component indicators, followed by a decision on using additive or geometric aggregation, and there is little agreement or scientific guidance on appropriate weighting techniques (Sharpe 2004). Again, these are normative decisions (Böhringer and Jochem 2007; Binder et al. 2010), despite the fact that methods can be objectively and transparently described, and the end result may appear arbitrary to end-users (Singh et al. 2009). For these reasons, 25 of the aggregate indices did not weight indicators at all, and nine indices specified that end-users must define the weights, which gives users some influence over the final scores.

**Table 3:** Typology of uses for freshwater indices

Use	Description
Benchmarking and monitoring	This is often described as a primary way to “operationalize” sustainability. Indices provide a quantitative baseline of the status of a freshwater system, along with thresholds, and a means of monitoring changes or progress toward defined goals
Facilitating IWRM	Index development encourages, if not requires, an integration of knowledge from different sectors related to water. In this case, the emphasis of the index may be on supporting the implementation of IWRM principles (identifying data needs, facilitating cross-agency dialogue) as well as providing a composite picture where one did not previously exist. Strategic spatial planning is sometimes considered a sub-component of IWRM efforts
Prioritizing investment	Indices are commonly used to facilitate comparisons, whether across units (e.g., basins or countries) or among the sub-components of a particular index (e.g., “drinking water” vs. “sanitation”). They may highlight deficiencies that could benefit from strategic public investment, or they may offer a tool for private investors to minimize exposure to water-related risk
Public awareness	Indices summarize a substantial amount of data and information into a coherent “big picture,” and thus they are often useful as a public communication tool. This may be a primary goal for river basin organizations producing annual reports, or for researchers wanting to raise the profile of less visible aspects of freshwater social-ecological systems (e.g., a product’s water footprint)

Most of the indices we reviewed have been developed with a particular end-user in mind, which in turn determines the decision context (or primary use) and scale of application. In fact, 26 of the indices we reviewed were developed for specific end-users in specific regions, which may limit their application outside of those regions but should increase their salience compared to more generalized indices. Only twenty-eight indices in total included some form of consultation with likely end-users to either select or refine indicators, ranging from a questionnaire to participatory workshops. This suggests that most indices are relying more on a top-down approach, which may affect their uptake (Sala et al. 2015). By contrast, 11 indices made no clear reference to end-users besides suggesting that findings would be of interest to policymakers or the research community. Clearly, end-user groups such as civil society organizations and the general public have an interest in these sorts of indices as well, but among the indices we reviewed these groups are typically mentioned as secondary rather than primary audiences. The following sub-sections summarize the different types of end-users of these indices.

#### Local governments/utilities

One-fifth (*n* = 19) of the indices we reviewed were developed primarily for “communities,” often a municipal entity or a class of user (water utilities, farmers). Life-cycle assessments were the most commonly applied framework within this group, perhaps as a way to connect communities with the global freshwater resources they impact (Hoff et al. 2014), although the LCA method has also been applied exclusively to a city’s regional water supply (Stoeglehner et al. 2011) as well as to model the throughput, or water metabolism, of cities (Lundin and Morrison 2002; Carden and Armitage 2013). One of the most often referenced indices, the Water Poverty Index, was developed to be used at the local community scale (Sullivan et al. 2003) and, due to its relatively basic data requirements, is easily applied at the country level as well, although doing so can mask important sub-national variation (Sullivan et al. 2006).

#### Resource managers

About a third of the indices were developed to support regional assessments. This may reflect the increasing recognition of a need to plan for and manage water resources at the basin scale, although this group also includes indices developed to measure ecological health and not necessarily the interplay between ecosystem services and socioeconomic demand. A small subset of indices (Chaves and Alipaz 2007; Davies et al. 2010; Jun et al. 2011; Pandey et al. 2011; Corrêa and do Nascimento Teixeira 2013) identify their primary end-user group as river basin organizations, but the vast majority of indices make more general references to “water resource managers” or simply “resource managers.” This category is distinguished from the previous category through an emphasis on the resource itself as the object of assessment, either a particular water body or resource in a particular basin, which often incorporates multiple dependent human and ecological (including terrestrial) communities. End-users may be a heterogeneous mix of local community representatives, multiple types of water users, and national agencies, but all aligned as stakeholders within a basin or regional group of watersheds. The two primary uses for indices in this category are benchmarking and supporting IWRM efforts. Three of these indices (Cai et al. 2002; Jun et al. 2011; Sandoval-Solis et al. 2011) specifically cite their use as a tool to assist regional stakeholders in evaluating tradeoffs among competing water uses.

#### National policymakers

Water resource management may be local and regional, but national policies often dictate its implementation. Sixteen of the indices were oriented toward national policymakers or the Ministries that enact policies and manage resources. However, a large majority (*n* = 12) of these indices involved assessments at a sub-national scale, usually using basin boundaries—four of the indices were demonstrated using only a basin rather than a full country-scale assessment (Sullivan 2010; Perez-Foguet and Garriga 2011; Storer et al. 2011; Speed et al. 2012); in these cases, however, the authors emphasize that the indices should be scaled up to inform national policymakers. This scaling up of sub-national assessments then facilitates a comparative analysis: whether to map “water poor” populations (Perez-Foguet and Garriga 2011) or evaluate the performance of river basin organizations within a country (Hooper 2010).

#### International organizations

Signaling the global importance of water resource sustainability, 10 indices have been developed for international organizations or development agencies. Some of these indices were commissioned specifically by the end-user. These include globally comprehensive assessments like the Global Environment Facility’s Transboundary Watershed Assessment Program’s (TWAP) indices for river basins (UNEP and UNEP-DHI 2015), lakes (ILEC 2011), and aquifers (UNESCO-IHP et al. 2012), as well as project-scale methodologies such as the Sustainability Index of WASH Interventions (Lockwood 2010) supported by the U.S. Agency for International Development. Donor organizations are seeking to shift their development support from direct service provision (e.g., infrastructure) to long-term systemic issues such as financial sustainability or the equitable distribution of and access to these services, and indices provide a way to measure progress toward these goals.

#### Private sector

Only three indices were developed specifically for a corporate decision maker, all using a “hazard identification” analytical lens and are intended to help prioritize investments and mitigate “corporate water risk.” Despite these commonalities, each index in this category operates distinctly. The Aqueduct Water Risk Atlas and online tool (Gassert et al. 2014) offer maps of 12 global indicators, including three indicators that make up the component of “Regulatory and Reputational Risk,” capturing the unique concerns of corporate decision makers with regard to water. The World Business Council for Sustainable Development’s Global Water Tool (WBCSD 2015) allows users to input information on production sites (as well as supply chains) and then assess corporate exposure to risk based on country- and basin-specific data and projections for water stress. The tool’s outputs are also made to be consistent with corporate disclosure protocols such as the Global Reporting Initiative. The Global Water Tool was also used to assess 48 global companies as input to the Water Risk Filter developed by the German Investment Corporation (DEG), and WWF (Orr et al. 2011), the major difference being that the Water Risk Filter is oriented toward investors, who can use the tool and its indicators to screen for water-related risks among their investments.

## Discussion

There is no “one size fits all” approach most suitable for decision support, but our analysis points to certain strengths and weaknesses of the various approaches, in their normative and systemic dimensions (analytical lenses and indicator selection) and their procedural dimension (stakeholder involvement). One strength of the DPSIR and System Sustainability approaches, for example, is their deliberate attention to the linkages and dynamics among indicators, recognizing that sustainability is more than an aggregation of important issues (Singh et al. 2009). Some lenses focus on single elements of what are complex, context-specific interactions within social-ecological systems (Armitage et al. 2015); these indices miss the links to other elements (sub-systems), although they may be helpful in assessing heretofore less understood issues (e.g., water governance). There will always be an audience for simple or narrowly focused indicators, and so the question is whether such indicators are fit for the purpose of implementing IWRM. Conversely, there are limits to the degree of complexity that can be reduced to and represented by quantitative indicators.

While this review was constrained to water-related indices, we believe these insights are more widely applicable and, hopefully useful, to the general area of quantitative assessments of social-ecological systems. Although most indices appeal to general concepts of sustainability, a variety of end-users invariably have differing goals and ways of operationalizing the concept, which are influenced by the analytical lens that guides indicator selection. While it is not feasible or productive to rank and rate this diverse set of indices, we have distilled what we consider to be positive attributes of index development, some gaps that should be addressed, and some guidance on future index development and application. Scientific information is central to solving water-related challenges, but it is just one input into decision making (Armitage et al. 2015). We see an opportunity to improve the decision relevance of indices, but achieving this will require more involvement of end-users in problem definition and indicator selection. It also requires more consideration of proactive uses of indices, including forecasting, identifying and mitigating tradeoffs, and enhancing positive impacts (Sala et al. 2015).

### Decision relevance of indices

Many of the indices we reviewed have been developed as decision-support tools—this is evidenced particularly by the 28 indices where end-users helped select indicators to suit their informational needs and administrative mandate. Yet more than 70 % of indices did not formally consult end-users as part of the index development process, which leads to ambiguity in terms of how those indices are to be applied and whether they are fit for a particular purpose (Sala et al. 2015). Even in the field of freshwater life-cycle analysis, which has heretofore been oriented toward public awareness, researchers are advocating for the method to become a “first base for strategic decisions” (Kounina et al. 2012). In other words, indices should not be used merely to tell us how we are doing, but to help us determine necessary steps to sustain the freshwater systems we rely on. To close the implementation gap between IWRM principles and practice, decision makers need guideposts to help them set tangible and relevant goals, measure their progress, and course-correct as needed.

To be decision relevant, index development should begin with end-users transparently identifying objectives, followed by the selection of indicators that can assist in meeting the objectives. The recently developed “service delivery” and “institutional performance” indices offer good examples of goal-driven indices, as they have been developed to take a more systemic view of the long-term viability of aid interventions (Lockwood 2010) and water resource management arrangements (Hooper 2010), respectively. It is surprising that ecosystem services do not feature more prominently among the indices we reviewed, despite acknowledgment of a need for quantifying water’s range of contributions to human well-being (Seager 2001; Cosgrove and Loucks 2015), suggestions that humans should be at the center of freshwater assessments (Meyer 1997; Vugteveen et al. 2006), and the emphasis that ecosystem service analysis places on quantification. Cook and Spray (2012) argue that the ecosystem services concept, with its scientific grounding, may offer an opportunity to operationalize principles of IWRM. Many analysts have suggested that the lack of standardized classification schemes for ecosystem services has hampered the concept’s uptake among resource managers (Polasky et al. 2015; Shapiro et al. 2015), and there remain methodological challenges in measuring flows and actual demand for some services (Bagstad et al. 2014; Burkhard et al. 2014). Nevertheless, this is a promising research avenue that could support useful new indicators. The link between freshwater ecological health and the services a healthy ecosystem can deliver deserves more attention. Measuring this link would connect environmental degradation (or improvement) more directly to changes in human well-being, thus also linking natural resource management with decisions surrounding infrastructure service delivery.

Most existing indices either highlight problems or refine our understanding of complex challenges, and are generally insufficient to provide insight into policies for improving conditions (Srinivasan et al. 2012) even if that is their purported aim. Risk assessment is the most common analytical lens used to construct indices (representing 25 % of indices), and tends to rely on indicators that focus on environmental stressors and exposure to hazards. Life-cycle analysis (9 % of indices) associates these stressors with a process or place. The DPSIR analytical lens (used in 13 % of reviewed indices) similarly focuses on environmental pressures and degradation, as do the 16 % of indices based on ecological health as an analytical lens. Understanding stressors is necessary but insufficient, as IWRM involves the full range of interactions between human and freshwater systems and thus frames challenges as optimizing long-term benefits for current and future generations. Such goals are inherently complex, involve large uncertainties, and require decision makers to navigate tradeoffs between water delivery and consumption, ecosystem services and ecosystem function and beneficiary groups. This calls for integrating knowledge from multiple disciplines, and in the case of indicators, it also requires attempts to measure phenomena and concepts that are hardly settled within the scientific community (Hester and Little 2013). Challenging as this may be, we believe indices can and must move in this direction if they are to support implementation of IWRM principles.

### Scenarios, tradeoffs, and thresholds

Researchers and policymakers increasingly recognize the benefits of incorporating scenario analysis alongside baseline assessments (2030 Water Resources Group 2009; Sullivan 2010; Doczi 2014), whether as additional indicators (UNEP and UNEP-DHI 2015) or as inputs to assess projected index values for the future (Devineni et al. 2013). Scenario analysis is important in order to incorporate a range of possible impacts from climate change, for example, but such scenarios should also factor in shifts in agricultural practices, infrastructure and industrial development, population growth, and land use change, all of which may, depending on the scale of analysis, have an appreciable impact on freshwater resources, and can be modeled with more certainty (and over shorter timescales) than climate change. A majority (*n* = 51) of the indices we reviewed are technically fit to support scenario analysis. By technically fit, we mean that these indices either measure biophysical and/or socioeconomic indicators with conceptual foundations for quantitative modeling. Better integration of these models, by combining hydrologic models with land use, vegetation, climate, and socioeconomic models, is a promising area for research (Vogel et al. 2015), and one that could support further applications of water-related indices.

Scenario analysis would facilitate identification and evaluation of tradeoffs and synergies among water demands and related ecosystem services. Nearly one-third (*n* = 28) of the indices we reviewed make reference to these sorts of tradeoffs within their documentation, most commonly in the context of water allocation among competing demands. But far fewer (*n* = 11) offer suggestions on how the index can be used to help evaluate tradeoffs, primarily the spatial allocation of water supply. Tradeoffs, synergies, and their implications are an essential part of the debate around sustainability and water resource management (Loucks 1997). Historically, humans have pursued some form of water “security” with limited regard for these tradeoffs (Pahl-Wostl et al. 2013). Tradeoffs imply losing something in order to gain something else, and so a better understanding of water-related tradeoffs could also help steer discussions beyond risk reduction and “least possible manipulation” toward a goal of the “best possible manipulation” of freshwater systems (Falkenmark 2003). This necessitates further discussion and negotiation about who or what is benefitting from freshwater services, but these conversations could be aided by indices that illustrate a vision (or competing visions) for the future. The concept of tradeoffs and synergies overlaps considerably with the presently *en vogue* water-food-energy nexus (Gupta et al. 2013) which, while conceptually intriguing, is another step removed from the realities of sectoral management and decision making. It also does not reflect that water is often the limiting factor, at least at a basin scale (Walmsley et al. 2001); food and energy are not geographically constrained in the same way that water resources are, and can be adequately represented as competing demands within a freshwater social-ecological system, rather than requiring a new field of nexus indicators.

Further exploration of tradeoffs would also force researchers (and end-users) to give greater consideration to the thresholds on individual indicators below or above which might signal unacceptable changes, and the potentially non-linear relationships between changes in normative indicators and the values humans place on them (Heink and Kowarik 2010). For example, the widely used indices of biotic integrity set a reference point for the ecological function of water bodies, but do not address the question of how much of a decline from this reference point constitutes a threat to either the ecosystem or the human communities that may value it (Beck and Hatch 2009). Assessments require such reference points to aid in interpretation, but this is a subjective exercise (Heink and Kowarik 2010) that is typically left to experts or policymakers. Some thresholds, such as Falkenmark’s indicator, are widely accepted but are arbitrary decisions rather than scientific facts. Rockström et al.’s (2009b) “planetary boundary” for water resources (4000 km^3^ consumed per year) may have already been surpassed, counter to the authors’ estimate of current use being only 2600 km^3^ and thus highlighting the major uncertainties in such calculations (Jaramillo and Destouni 2015), while others, such as pollution concentrations that affect changes in ecosystem processes, have a basis in scientific fact. Many of the thresholds relating to water scarcity suffer the problem of being developed top-down without accounting for locally variable conditions and institutions (Srinivasan et al. 2012) or local ecology (Smakhtin et al. 2004). We argue that basin-scale assessments involving stakeholder and scientific input (e.g., for goal-setting, data collection, scenario development, and weighting) are needed for progress in understanding these tradeoffs and what appropriate thresholds might be.

### Balancing salience, legitimacy, and credibility

The continued development of indices suggests that there is a need for new and improved ways to assess freshwater systems, as well as a need to synthesize the complex information that informs our understanding of them (2030 Water Resources Group 2009). As the stressors on freshwater systems increase in magnitude and abundance, informational needs have become diverse and more nuanced. Furthermore, watershed governance occurs at multiple spatial scales (Sullivan et al. 2006; Parkes et al. 2010). Informational needs (and the ability to manage freshwater systems) change as the spatial scale moves from a local to global community. Rather than scaling up or down, indices ought to clearly define their niche, whether it is global awareness raising or basin-scale decision support.

We recommend that water indices strive to reach a balance across salience, legitimacy, and credibility, recognizing that these attributes are often closely linked (Cash et al. 2003; Armitage et al. 2015). These three criteria should foster greater relevance of indices to address the social and ecological implications of water use across all sectors; the development and application of indices needs to consider the objectives of policy and decision makers in the context of the information gained from the physical, natural, and social sciences (Turnhout et al. 2007). Salience is achieved by providing information that is useful to those who can act on that information. Global indices and assessments, for example, are of limited use to the communities managing freshwater resources (Rijsberman 2006) because they are restricted to globally uniform datasets, often at a coarse spatial resolution, and are unable to reflect differentiated local or regional informational needs, interests, and capacities to respond (Srinivasan et al. 2012). Basin-scale assessments might be the most appropriate scale for index application, as they can also be aggregated to the national level to provide information relevant to national policymakers. Water footprinting techniques are also moving in this direction, incorporating finer scale regionalized assessments (including indices) as input “characterization factors” in impact assessments (Kounina et al. 2012). But even basin-scale indices might not be salient if they are developed with too little input from end-users. Most indices’ documentation not only include suggestions that their overarching framework can be adapted to local needs or data constraints, and this is obvious, but also diminishes the value of the initial framework if it must be dismantled.

Legitimacy derives from the perception that an index respects divergent values and that it has been developed in an unbiased way. Several indices under the “System Sustainability” analytical lens category combine social, economic, and environmental indicators as a way of conferring legitimacy among divergent interests. But moving too far in this direction, of arbitrarily including indicators without demonstrating their links to one another, may limit the salience of integrating them in a single index approach. Legitimacy is not achieved by being inclusive in terms of the indicators themselves, but by offering an inclusive process for developing the index or allowing some flexibility for adapting the index to local circumstances. A simple but often overlooked step within these indices that could enhance their legitimacy is leaving the weighting and aggregation decisions to stakeholders themselves. While there is no universal agreement on whether to aggregate the output of indicators, aggregation techniques abound in the literature (the analytic hierarchy process (Saaty 2005) appearing to be the most popular technique employed in the indices we reviewed) for soliciting stakeholder input into combining information within indices and across stakeholders. But more generally, indices that incorporate participatory approaches to defining issues and refining indicators are likely to be perceived as more legitimate (Wissen Hayek et al. 2016) and thus more likely to be adopted.

Finally, credibility, or the scientific and technical rigor of an index, has been the primary focus within the research community, but it does not address the challenge of bridging science and policy. Decision theoretic frameworks, that distinguish between the roles that scientific information and social values play in decisions, are well poised to address this but have not been utilized to their full potential in the application of indices to water resource use. Rather than isolate variables in order to “maintain” scientific credibility, researchers and stakeholders, across multiple facets, should work together to ensure indices are salient, legitimate and credible to provide a better integration of the social and ecological aspects of freshwater systems. In this regard, we see the Sustainable System analytical lens as a promising area for further development, with its emphasis on linkages among variables and human dependence on resources.

### Future directions

It is clear from the proliferation of water-related indices over the last two decades that there is a need for assessments of freshwater systems, and an improving capability to tailor these assessments to the informational needs of different end-users. There will always be competing demands on water resources, and in much of the world these demands (and stressors) are increasing. These have come to the forefront with the looming and current freshwater crisis, and climate change could exacerbate existing tensions. Synthetic ways to measure and balance all of the needs and uses of water resources are essential to understanding and managing freshwater resources and their stressors. Across the indices we reviewed, most focus on water as a scarce or highly demanded resource, and the majority of indices explicitly account for human dependence on this resource. There are indices that focus on the health of the freshwater ecosystem that is the source of the services we depend on, but they stop short of assessing these services, or quantifying the relationship between ecosystem health and service delivery. Moreover, none of the indices we reviewed fully assess the tradeoffs inherent in integrating land and water resource management to optimize benefits. Therefore, there is an imperative for analysis of both the supply and demand components of freshwater systems and the resulting impacts to the ecosystems that provide freshwater. This will require indices that can measure physical and biological properties, the needs of freshwater ecosystems as well as societal needs. Indices on their own will not immediately make clear the decisions managers need to make to ensure sustainability and equitable distribution of water resources, but they provide crucial information to informing such decisions if they can span both the scientific and social attributes of water resources. Ultimately, they should orient us toward sustaining our most critical natural resource.

## Electronic supplementary material

Below is the link to the electronic supplementary material.

Supplementary material 1 (PDF 217 kb)
