# Supplementary material for: Assessing the sustainability of freshwater systems: A critical review of composite indicators
Source: Ambio. 2016 Jun 1;45(7):765–80. doi: 10.1007/s13280-016-0792-7 (PMC5055483; doi:10.1007/s13280-016-0792-7)
Supplement: Supplementary file 1 — Supplementary material 1 (PDF 217 kb) [file 13280_2016_792_MOESM1_ESM.pdf]

Ambio

Electronic Supplementary Material
This supplementary material has not been peer reviewed.

Title: **Assessing the sustainability of freshwater systems: A critical review of composite indicators**

Authors: Derek Vollmer, Helen M. Regan, Sandy J. Andelman

Table S1 List of indices reviewed, their analytical lenses and intended use.

Project/Index name	Source	Analytical lens	Intended end-user	Primary use
Adaptive Capacity Index*	Pandey et al. (2011)	Risk assessment	River basin organization	Prioritizing investment
Agricultural Water Poverty Index	Forouzani and Karami (2011)	Risk assessment	Agricultural water users	Public awareness
Aqueduct Water Risk Atlas	Gassert et al. (2014)	Risk assessment	Corporate	Prioritizing investment
Arab Water Sustainability Index*	Ali (2009)	Risk assessment	National policymakers	Benchmarking
Arctic Water Resource Vulnerability Index*	Alessa et al. (2008)	Risk assessment	Community managers	Benchmarking
Asian Water Development Outlook*	ADB and APWF (2013)	Risk assessment	National policymakers	Benchmarking
Assessing Ecosystem Services in the Goulburn Broken Catchment*	Abel et al. (2003)	Ecosystem services	Policy makers, planners, resource managers	IWRM
Bay Health Index*	Williams et al. (2009)	Ecological health	Resource managers	Benchmarking
Benthic Index of Biotic Integrity	Kerans and Karr (1994)	Ecological health	Resource managers	Benchmarking
Blue Water Sustainability Index	Wada and Bierkens (2014)	Risk assessment	Not defined	Public awareness
California Water Sustainability Indicators Framework*	Shilling (2013)	System sustainability	Resource managers	Benchmarking
Canada Water Sustainability Index (C-WSI)*	PRI (2007)	Risk assessment	National policymakers	IWRM
City Blueprints	van Leeuwen et al. (2012)	LCA	Local government/utilities	Benchmarking
Climate Vulnerability Index (CVI)	Sullivan and Meigh (2005)	Risk assessment	National policymakers	Prioritizing investment
Corporate Responsibility Performance Indicators	Staben et al. (2010)	System sustainability	Water supply companies	Reporting
Cultural Health Index for Streams and Waterways*	Tipa and Teirney (2006)	Ecosystem services	National policymakers	IWRM
Cumulative threat indices	Vorosmarty et al. (2010)	Risk assessment	Not defined	Public awareness
Ecosystem Health Index Methodology	Xu et al. (2005)	Ecological health	Resource managers	Benchmarking
Enhanced Water Poverty Index (eWPI)	Perez-Foguet and Garriga (2010)	DPSIR	National policymakers	IWRM
EU Water Framework Directive Indicators	Nixon et al. (2003)	DPSIR	National policymakers	Benchmarking
Fish-based index for the assessment of river health	Oberdorff et al. (2002)	Ecological health	Resource managers	Benchmarking
Flood Resilience Index (FRI)	Kotzee et al. (2016)	Risk assessment	Local government/utilities	Prioritizing investment
Framework for Assessing River and Wetland Health*	Storer et al. (2011)	Ecological health	Ministries	Benchmarking
Framework for Measuring Sustainable Development in Catchment Systems	Walmsley (2002)	DPSIR	National policymakers	IWRM
Framework for Sustainability Analysis in WRM	Cai et al. (2002)	System sustainability	Resource managers	IWRM

Framework of Sustainable Water Resource Management Indicators	Liaw et al. (2000)	DPSIR	Resource managers	Benchmarking
Fraser Basin Sustainability Indicators*	FBC (2011)	System sustainability	Public	Public awareness
Freshwater Health Assessment (FHA)*	WWF Canada (2013)	Ecological health	Resource managers	Benchmarking
Freshwater Provisioning Index for Humans	Green et al. (2015)	Risk assessment	Not defined	Prioritizing investment
Groundwater Resources Sustainability Indicators	Vrba et al. (2007)	DPSIR	National policymakers	Benchmarking
H2Oe	Ridoutt and Pfister (2013)	LCA	Not defined	Benchmarking
Human Impact on Freshwater Ecosystem Services	Dodds et al. (2013)	Ecosystem services	Not defined	Benchmarking
Index of Biotic Integrity	Karr (1981)	Ecological health	Resource managers	Benchmarking
Indicators for assessment of Integrated Lake Basin Management*	Chidamodzi and Muhandiki (2015)	Institutional performance	Resource managers	IWRM
Indicators of Hydrologic Alteration	Richter et al. (1996)	Ecological health	Resource managers	Planning
Life cycle assessment for urban water systems	Lundin and Morrison (2002)	LCA	Urban environmental managers	Benchmarking
MCE of Water Resources Sustainability	Kang and Lee (2011)	System sustainability	Resource managers	Benchmarking
Measuring sustainability in Israel's water system*	Kay (2000)	Risk assessment	National policymakers	Benchmarking
Multi-regional input-ouput model	Ewing et al. (2012)	LCA	Not defined	Public awareness
Multiple taxonomic groups to index ecological condition of lakes	O'Connor et al. (2000)	Ecological health	Resource managers	Benchmarking
National Water Quality Index	Carr and Rickwood (2008)	Risk assessment	International organizations	Prioritizing investment
Performance Index for Assessing Urban Water Systems	Bagheri et al. (2006)	System sustainability	Local government/utilities	Benchmarking
Performance Indicators for Irrigation and Drainage	Bos (1997)	Infrastructure service delivery	Not defined	Benchmarking
Performance of RBOs*	Gallego-Ayala and Juizo (2012)	Institutional performance	River basin organization	Benchmarking
Planktonic Index of Biotic Integrity	Lacouture et al. (2006)	Ecological health	Resource managers	Benchmarking
Post-2015 WASH indicators	JMP/WHO/UNICEF (2015)	Infrastructure service delivery	Development agencies	Benchmarking
Rapid Appraisal Wetland Condition Index	Spencer et al. (1998)	Ecological health	Resource managers	Benchmarking
RBO Performance Indicators	Hooper (2010)	Institutional performance	National policymakers	Benchmarking
River Basin Health Indicators (RHA)*	Speed et al. (2012)	Ecological health	Ministries	Benchmarking
River Basin Sustainability Index	Sood and Ritter (2011)	Risk assessment	Resource managers	Benchmarking
Rural water supply sustainability indicators	Sara and Katz (2005)	Infrastructure service delivery	Development agencies	Benchmarking
Spatial Distribution of Water Resource Vulnerability in the Columbia River Basin*	Chang et al. (2013)	Risk assessment	Resource managers	IWRM
Spatial Water Resources Vulnerability Index	Jun et al. (2011)	DPSIR	River basin organization	IWRM

Spatially Distributed Water Stress Index*	Devineni et al. (2013)	Risk assessment	National policymakers	Prioritizing investment
Stream-Wetland-Riparian Index*	Brooks et al. (2009)	Ecological health	Resource managers	Benchmarking
Sustainability Assessment Tool for community-managed rural water systems	Schweitzer and Mihelcic (2013)	Infrastructure service delivery	Community managers	Benchmarking
Sustainability Index	Loucks (1997)	System sustainability	Not defined	IWRM
Sustainability Index for Integrated Urban Water Management*	Carden and Armitage (2013)	LCA	Urban environmental managers	IWRM
Sustainability Index for Water Resources Planning and Management	Sandoval-Solis et al. (2011)	System sustainability	Resource managers	IWRM
Sustainability Index of WASH Interventions	Lockwood (2010)	Infrastructure service delivery	Development agencies	Benchmarking
Sustainability Indicators for a small lake basin in Western Mexico*	Shear and Anda (2009)	DPSIR	Resource managers	Planning
Sustainability Indicators for WRM in Brazil*	Correa and Teixera (2013)	System sustainability	River basin organization	IWRM
Sustainability Wheel	Schneider et al. (2014)	Institutional performance	Regional decision makers	IWRM
Sustainable Rivers Audit*	Davies et al. (2010)	Ecological health	River basin organization	Benchmarking
SWITCH Indicators for Urban WM	van der Steen (2011)	DPSIR	Local government/utilities	Benchmarking
SWRR Indicator Framework	SWRR (2008)	System sustainability	National policymakers	Benchmarking
Tiered Framework for Assessing Groundwater Ecosystem Health	Korbel and Hose (2011)	Ecological health	Resource managers	Benchmarking
TWAP Groundwater	UNESCO-IHP et al. (2012)	DPSIR	International organizations	Prioritizing investment
TWAP Lakes	ILEC (2011)	DPSIR	International organizations	Prioritizing investment
TWAP River Basins	UNEP and UNEP-DHI (2015)	DPSIR	International organizations	Prioritizing investment
UN World Water Development Report	WWAP (2015)	DPSIR	International organizations	Benchmarking
Urban Water System Sustainability	Marques et al. (2015)	System sustainability	Local government/utilities	Benchmarking
Vulnerability Index (VI)*	Babel et al. (2011)	Risk assessment	Resource managers	IWRM
Water and Sanitation Index of Development Effectiveness	Stampini et al. (2012)	Infrastructure service delivery	International organizations	Benchmarking
Water and Sanitation Sustainability Index	Iribarnegaray et al. (2012)	System sustainability	Local government/utilities	Benchmarking
Water Footprint	Hoekstra et al. (2009)	LCA	Not defined	Benchmarking
Water Footprints of Cities	Hoff et al. (2014)	LCA	Local government/utilities	Public awareness
Water Impact Index	Bayart et al. (2014)	LCA	Local government/utilities	Public awareness
Water Impact Index (WiiX)	Veolia (2009)	LCA	Local government/utilities	Public awareness
Water Management Sustainability Indicators	Ioris et al. (2008)	System sustainability	Resource managers	IWRM
Water Management Towards Sustainability	Clausen and Hafkesbrink (2005)	Institutional performance	National policymakers	Influence national policy

Water Poverty Index (WPI)	Sullivan et al. (2003)	Risk assessment	Local government/utilities	Benchmarking
Water Resources Management Sustainability Index Tool	FIU-GLOWS (2014)	Institutional performance	Development agencies	Benchmarking
Water Risk Filter	Orr et al. (2011)	Risk assessment	Corporate	Prioritizing investment
Water Scarcity and Allocation Indices	Han et al. (2013)	Risk assessment	Resource managers	Planning
Water Scarcity Index	Smakhtin et al. (2004)	Risk assessment	Not defined	Public awareness
Water Stress Index	Pfister et al. (2009)	LCA	Not defined	Public awareness
Water Supply Footprint	Stoeglehner et al. (2011)	LCA	Local government/utilities	IWRM
Water Supply Sustainability Index (WSSI)	Tetra-Tech/NRDC (2010)	Risk assessment	National policymakers	Public awareness
Water Sustainability Indicators for Abu Dhabi*	Alsalmi et al. (2013)	DPSIR	Local government/utilities	Benchmarking
Water Use Benefit Index*	Smajgl et al. (2010)	Ecosystem services	Community managers	Benchmarking
Water Vulnerability Index (WVI)	Sullivan (2011)	Risk assessment	National policymakers	IWRM
Watershed Sustainability Index (WSI)	Chaves and Alipaz (2007)	DPSIR	River basin organization	IWRM
WBSCD Global Water Tool (GWT)	WBCSD (2015)	Risk assessment	Corporate	Prioritizing investment
West Java Water Sustainability Index*	Juwana et al. (2014)	System sustainability	Resource managers	IWRM

*developed for specific geographic area

Appendix S1 Reference list for indices included in review.

Abel, N., S. Cork, R. Gorddard, J. Langridge, A. Langston, R. Plant, W. Proctor, P. Ryan, et al. 2003. *Natural Values: Exploring options for enhancing ecosystem services in the Goulburn Broken Catchment*. Canberra, Australia. doi:ISBN 0 9580845 7 2, http://www.ecosystemservicesproject.org/html/publications/docs/nveo/Natural_Values.pdf.

ADB (Asian Development Bank) and APWF (Asia Pacific Water Forum). 2013. *Asian Water Development Outlook 2013: Measuring Water Security in Asia and the Pacific*. Mandaluyong City, Philippines: Asian Development Bank, http://www.adb.org/publications/asian-water-development-outlook-2013.

Alessa, L., A. Kliskey, R. Lammers, C. Arp, D. White, L. Hinzman, and R. Busey. 2008. The arctic water resource vulnerability index: an integrated assessment tool for community resilience and vulnerability with respect to freshwater. *Environ Manage* 42: 523–541. doi:10.1007/s00267-008-9152-0.

Ali, H. M. M. 2009. Development of Arab Water Sustainability Index Using Principal Component Analysis. In *Thirteenth International Water Technology Conference IWTC13*, 1563–1579. Hurghada, Egypt, http://www.iwtc.info/2009_pdf/19-1.pdf.

Alsalmi, H., H. Elkadi, and S. Leao. 2013. Urban growth in arid environments: Developing water sustainability indicators for Abu Dhabi (UAE). In *6th Making Cities Liveable Conference*. Melbourne, Victoria, Australia.

Babel, M. S., V. P. Pandey, A. A. Rivas, and S. M. Wahid. 2011. Indicator-based approach for assessing the vulnerability of freshwater resources in the Bagmati River basin, Nepal. *Environ Manage* 48: 1044–1059. doi:10.1007/s00267-011-9744-y.

Bagheri, A., A. Asgary, J. Levy, and M. Rafieian. 2006. A performance index for assessing urban water systems: A fuzzy inference approach. *Journal American Water Works Association* 98: 84–92.

Bayart, J.-B., S. Worbe, J. Grimaud, and E. Aoustin. 2014. The Water Impact Index: a simplified single-indicator approach for water footprinting. *International Journal of Life Cycle Assessment* 19: 1336–1344. doi:10.1007/s11367-014-0732-3.

Bos, M. G. 1997. Performance indicators for irrigation and drainage. *Irrigation and Drainage Systems* 11: 119–137. doi:10.1023/a:1005826407118.

Brooks, R., M. McKenney-Easterling, M. Brinson, R. Rheinhardt, K. Havens, D. O'Brien, J. Bishop, J. Rubbo, et al. 2009. A Stream-Wetland-Riparian (SWR) index for assessing condition of aquatic ecosystems in small watersheds along the Atlantic slope of the eastern US. *Environmental Monitoring and Assessment* 150: 101–117. doi:10.1007/s10661-008-0673-z.

Cai, X. M., D. C. McKinney, and L. S. Lasdon. 2002. A framework for sustainability analysis in water resources management and application to the Syr Darya Basin. *Water Resources Research* 38. doi:10.1029/2001wr000214.

Carden, K., and N. P. Armitage. 2013. Assessing urban water sustainability in South Africa – not just performance measurement. *Water SA* 39. doi:10.4314/wsa.v39i3.1.

Carr, G. M., and C. J. Rickwood. 2008. *Water Quality: Development of an index to assess country performance*. Gatineau, Ontario, Canada: UNEP GEMS/Water Programme, http://www.unep.org/gemswater/Portals/24154/pdfs/new/2008%20WQ%20Index%20development%20White%20Paper.pdf.

Chang, H., I.-W. Jung, A. Strecker, D. Wise, M. Lafrenz, V. Shandas, H. Moradkhani, A. Yeakley, et

al. 2013. Water Supply, Demand, and Quality Indicators for Assessing the Spatial Distribution of Water Resource Vulnerability in the Columbia River Basin. *Atmosphere-Ocean* 51: 339–356. doi:10.1080/07055900.2013.777896.

Chaves, H. M. L., and S. Alipaz. 2007. An integrated indicator based on basin hydrology, environment, life, and policy: the watershed sustainability index. *Water Resources Management* 21: 883–895.

Chidammodzi, C. L., and V. S. Muhandiki. 2015. Development of indicators for assessment of Lake Malawi Basin in an Integrated Lake Basin Management (ILBM) framework. *International Journal of the Commons* 9: 209–236.

Clausen, H., and J. Hafkesbrink. 2005. Water Management Towards Sustainability - An Indicator System to Assess Innovations. In *Indicator Systems for Sustainable Innovation*, ed. J. Horbach, 179–203. Heidelberg, Germany: Physica-Verlag. doi:10.1007/3-7908-1620-5_9.

Corrêa, M. A., and B. A. do N. Teixeira. 2013. Developing sustainability indicators for water resources management in Tietê-Jacaré Basin, Brazil. *Journal of Urban and Environmental Engineering* 7: 8–14. doi:10.4090/juee.2013.v7n1.008014.

Davies, P. E., J. H. Harris, T. J. Hillman, and K. F. Walker. 2010. The Sustainable Rivers Audit: assessing river ecosystem health in the Murray-Darling Basin, Australia. *Marine and Freshwater Research* 61: 764–777. doi:10.1071/mf09043.

Devineni, N., S. Perveen, and U. Lall. 2013. Assessing chronic and climate-induced water risk through spatially distributed cumulative deficit measures: A new picture of water sustainability in India. *Water Resources Research* 49: 2135–2145. doi:10.1002/wrcr.20184.

Dodds, W. K., J. S. Perkin, and J. E. Gerken. 2013. Human Impact on Freshwater Ecosystem Services: A Global Perspective. *Environmental Science & Technology* 47: 9061–9068. doi:10.1021/es4021052.

Ewing, B. R., T. R. Hawkins, T. O. Wiedmann, A. Galli, A. E. Ercin, J. Weinzettel, and K. Steen-Olsen. 2012. Integrating ecological and water footprint accounting in a multi-regional input-output framework. *Ecological Indicators* 23: 1–8. doi:10.1016/j.ecolind.2012.02.025.

FBC (Fraser Basin Council). 2011. *Measuring & Reporting on Sustainability: A Report on Lessons Learned*. Fraser Basin Council, http://www.fraserbasin.bc.ca/_Library/Comm_Indicators/report_indicators_lessons_2011.pdf.

FIU-GLOWS (Florida International University- Global Water for Sustainability Program). 2014. Water Resources Management Sustainability Index Tool (WRM SIT) Initiative-- Terms of Reference.

Forouzani, M., and E. Karami. 2011. Agricultural water poverty index and sustainability. *Agronomy for Sustainable Development* 31: 415–431. doi:10.1051/agro/2010026.

Gallego-Ayala, J., and D. Juizo. 2012. Performance evaluation of River Basin Organizations to implement integrated water resources management using composite indexes. *Physics and Chemistry of the Earth* 50-52: 205–216. doi:10.1016/j.pce.2012.08.008.

Gassert, F., M. Luck, M. Landis, P. Reig, and T. Shiao. 2014. *Aqueduct Global Maps 2.1: Constructing Decision-Relevant Global Water Risk Indicators*. *Working Paper*. Washington, DC: World Resources Institute, http://www.wri.org/publication/aqueduct-global-maps-21.

Green, P. A., C. J. Vörösmarty, I. Harrison, T. Farrell, L. Sáenz, and B. M. Fekete. 2015. Freshwater ecosystem services supporting humans: Pivoting from water crisis to water solutions. *Global Environmental Change* 34: 108–118. doi:10.1016/j.gloenvcha.2015.06.007.

Han, M., Q. Ren, Y. Wang, J. Du, Z. Hao, F. Sun, L. Cheng, S. Qi, et al. 2013. Integrated Approach to Water Allocation in River Basins. *Journal of Water Resources Planning and Management-*

ASCE 139: 159–165. doi:10.1061/(asce)wr.1943-5452.0000255.
Hoekstra, A. Y., A. K. Chapagain, M. M. Aldaya, and M. M. Mekonnen. 2009. *Water Footprint Manual: State of the Art 2009*. Enschede, The Netherlands: Water Footprint Network, http://waterfootprint.org/media/downloads/WaterFootprintManual2009.pdf.
Hoff, H., P. Döll, M. Fader, D. Gerten, S. Hauser, and S. Siebert. 2014. Water footprints of cities – indicators for sustainable consumption and production. *Hydrology and Earth System Sciences* 18: 213–226. doi:10.5194/hess-18-213-2014.
Hooper, B. 2010. River basin organization performance indicators: application to the Delaware River basin commission. *Water Policy* 12: 461–478. doi:10.2166/wp.2010.111.
ILEC. 2011. *Methodology for the Assessment of Transboundary Lake Basins. Volume 3. Methodology for the GEF Transboundary Waters Assessment Programme*. Nairobi: UNEP, 69pp., http://www.unep.org/dewa/Portals/67/pdf/TWAP-Volume-3-Methodology_for_Lake_Basins-low-res.pdf.
Ioris, A. A. R., C. Hunter, and S. Walker. 2008. The development and application of water management sustainability indicators in Brazil and Scotland. *Journal of environmental management* 88: 1190–1201. doi:10.1016/j.jenvman.2007.06.007.
Iribarnegaray, M. A., F. R. Copa, M. L. Gatto D'Andrea, M. F. Arredondo, J. D. Cabral, J. J. Correa, V. I. Liberal, and L. Seghezzo. 2012. A comprehensive index to assess the sustainability of water and sanitation management systems. *Journal of Water Sanitation and Hygiene for Development* 2: 205–222. doi:10.2166/washdev.2012.005.
JMP (WHO/UNICEF Joint Monitoring Programme). 2015. Post-2015 WASH targets and indicators, http://www.who.int/water_sanitation_health/monitoring/coverage/wash-post-2015-rev.pdf?ua=1.
Jun, K. S., E. S. Chung, J. Y. Sung, and K. S. Lee. 2011. Development of spatial water resources vulnerability index considering climate change impacts. *Science of the Total Environment* 409: 5228–5242. doi:10.1016/j.scitotenv.2011.08.027.
Juwana, I., N. Muttil, and B. J. C. Perera. 2014. Application of West Java water sustainability index to Citarum catchment in West Java, Indonesia. *Water Science & Technology: Water Supply* 14: 1150. doi:10.2166/ws.2014.075.
Kang, M.-G., and G.-M. Lee. 2011. Multicriteria evaluation of water resources sustainability in the context of watershed management. *Journal of the American Water Resources Association* 47: 813–827. doi:10.1111/j.1752-1688.2011.00559.x.
Karr, J. R. 1981. Assessment of biotic integrity using fish communities. *Fisheries* 6: 21–27.
Kay, P. A. 2000. Measuring sustainability in Israel's water system. *Water International* 25: 617–623.
Kerans, B. L., and J. R. Karr. 1994. A Benthic Index of Biotic Integrity (B-IBI) for Rivers of the Tennessee Valley. *Ecological Applications* 4. Ecological Society of America: 768–785. doi:10.2307/1942007.
Korbel, K. L., and G. C. Hose. 2011. A tiered framework for assessing groundwater ecosystem health. *Hydrobiologia* 661: 329–349. doi:10.1007/s10750-010-0541-z.
Kotzee, I., and B. Reyers. 2016. Piloting a social-ecological index for measuring flood resilience: A composite index approach. *Ecological Indicators* 60: 45–53. doi:10.1016/j.ecolind.2015.06.018.
Lacouture, R. V, J. M. Johnson, C. Buchanan, and H. G. Marshall. 2006. Phytoplankton index of biotic integrity for Chesapeake Bay and its tidal tributaries. *Estuaries and Coasts* 29. Springer-Verlag: 598–616. doi:10.1007/BF02784285.
van Leeuwen, C. J., J. Frijns, A. van Wezel, and F. H. M. van de Ven. 2012. City Blueprints: 24 Indicators to Assess the Sustainability of the Urban Water Cycle. *Water Resources Management* 26: 2177–2197. doi:10.1007/s11269-012-0009-1.

Liaw, C.-H., C.-C. Cheng, and C.-K. Hsieh. 2000. A framework of sustainable water resources management indicators. *Journal of the Chinese Institute of Environmental Engineering* 10: 311–322.
Lockwood, H. 2010. *Sustainability Index of WASH Interventions: Global Findings and Lessons Learned*. USAID and Rotary International, http://www.washplus.org/sites/default/files/WashSustainabilityIndex.pdf.
Loucks, D. P. 1997. Quantifying trends in system sustainability. *Hydrological Sciences Journal* 42: 513–530. doi:10.1080/02626669709492051.
Lundin, M., and G. M. Morrison. 2002. A life cycle assessment based procedure for development of environmental sustainability indicators for urban water systems. *Urban Water* 4: 145–152.
Marques, R. C., N. F. da Cruz, and J. Pires. 2015. Measuring the sustainability of urban water services. *Environmental Science & Policy* 54: 142–151. doi:10.1016/j.envsci.2015.07.003.
Nixon, S., Trent, Z., Marcuello, C., Lallana, C., 2003. Europe's Water: An Indicator- based Assessment. European Environment Agency, Copenhagen, http://www.eea.europa.eu/publications/topic_report_2003_1.
O'Connor, R. J., T. E. Walls, and R. M. Hughes. 2000. Using multiple taxonomic groups to index the ecological condition of lakes. *Environmental Monitoring and Assessment* 61: 207–228. doi:10.1023/a:1006119205583.
Oberdorff, T., D. Pont, B. Hugueny, and J. P. Porcher. 2002. Development and validation of a fish-based index for the assessment of "river health" in France. *Freshwater Biology* 47: 1720–1734. doi:10.1046/j.1365-2427.2002.00884.x.
Orr, S., R. Sánchez-Navarro, G. Schmidt, R. Seiz-Puyuelo, K. Smith, and J. Verberne. 2011. *Assessing Water Risk: A Practical Approach for Financial Institutions*. Berlin, Germany: WWF Germany, 59pp., https://www.deginvest.de/DEG-Englische-Dokumente/PDFs-Download-Center/DEG-WWF_Water_Risk.pdf.
Pandey, V. P., M. S. Babel, S. Shrestha, and F. Kazama. 2011. A framework to assess adaptive capacity of the water resources system in Nepalese river basins. *Ecological Indicators* 11: 480–488. doi:10.1016/j.ecolind.2010.07.003.
Perez-Foguet, A., and R. G. Garriga. 2011. Analyzing Water Poverty in Basins. *Water Resources Management* 25: 3595–3612. doi:10.1007/s11269-011-9872-4.
Pfister, S., A. Koehler, and S. Hellweg. 2009. Assessing the Environmental Impacts of Freshwater Consumption in LCA. *Environmental Science & Technology* 43: 4098–4104.
PRI (Policy Research Initiative). 2007. *Canadian Water Sustainability Index (CWSI) Project Report*. Government of Canada, http:// publications.gc.ca/Collection/PH4-38-2007E.pdf.
Richter, B. D., J. V. Baumgartner, J. Powell, and D. P. Braun. 1996. A method for assessing hydrologic alteration within ecosystems. *Conservation Biology* 10: 1163-1174.
Ridoutt, B. G., and S. Pfister. 2013. A new water footprint calculation method integrating consumptive and degradative water use into a single stand-alone weighted indicator. *International Journal of Life Cycle Assessment* 18: 204–217. doi:10.1007/s11367-012-0458-z.
Sandoval-Solis, S., D. C. McKinney, and D. P. Loucks. 2011. Sustainability Index for Water Resources Planning and Management. *Journal of Water Resources Planning and Management* 137: 381–390. doi:10.1061/(ASCE)WR.1943-5452.0000134.
Sara, J., and T. Katz. 2005. *Making Rural Water Supply Sustainable: Report on the Impact of Project Rules*. Edited by UNDP-World Bank Water and Sanitation Program. Washington, DC, USA, 87pp., http://documents.worldbank.org/curated/en/2005/01/6180881/making-rural-water-

supply-sustainable-report-impact-project-rules.
Schneider, F., M. Bonriposi, O. Graefe, K. Herweg, C. Homewood, M. Huss, M. Kauzlaric, H. Liniger, et al. 2014. Assessing the sustainability of water governance systems: the sustainability wheel. *Journal of Environmental Planning and Management*: 1–24. doi:10.1080/09640568.2014.938804.
Schweitzer, R. W., and J. R. Mihelcic. 2012. Assessing sustainability of community management of rural water systems in the developing world. *Journal of Water Sanitation and Hygiene for Development* 2: 20–30. doi:10.2166/washdev.2012.056.
Shear, H., and J. de Anda. 2009. Preliminary selection of sustainability indicators for a small lake basin in Western Mexico. *Local Environment* 14: 557–574. doi:10.1080/13549830902904144.
Shilling, F. 2013. *The California Water Sustainability Indicators Framework: Draft Final Report. Report to the California Department of Water Resources,* 231pp., http://www.waterplan.water.ca.gov/docs/cwpu2013/Final/vol4/sustainability/03CA_Water_Sustainability_Indicators_Assesment.pdf.
Smajgl, A., S. Larson, B. Hug, and D. M. De Freitas. 2010. Water use benefit index as a tool for community-based monitoring of water related trends in the Great Barrier Reef region. *Journal of Hydrology* 395: 1–9. doi:10.1016/j.jhydrol.2010.09.007.
Smakhtin, V., C. Revenga, and P. Döll. 2004. *Taking Into Account Environmental Water Requirements in Global-scale Water Resources Assessments. Comprehensive Assessment of Water Management in Agriculture*. Vol. 2. Colombo, Sri Lanka: International Water Management Institute, http://www.iwmi.cgiar.org/assessment/files/pdf/publications/ResearchReports/CARR2.pdf.
Sood, A., and W. F. Ritter. 2011. Developing a framework to measure watershed sustainability by using hydrological/water quality model. *Journal of Water Resource and Protection* 3: 788–804.
Speed, R., C. Gippel, N. Bond, S. Bunn, X. Qu, Y. Zhang, W. Liu, and X. Jiang. 2012. *Assessing river health and environmental flow requirements in Chinese rivers*. Brisbane, Australia: International Water Centre, http://watercentre.org/portfolio/rhef/attachments/technical-reports/assessment-of-river-health-and-eflow-requirements-in-chinese-rivers.
Spencer, C., A. I. Robertson, and A. Curtis. 1998. Development and testing of a rapid appraisal wetland condition index in south-eastern Australia. *Journal of environmental management* 54: 143–159. doi:10.1006/jema.1998.0212.
Staben, N., A. Hein, and T. Kluge. 2010. Measuring sustainability of water supply: performance indicators and their application in a corporate responsibility report. *Water Science and Technology: Water Supply* 10: 824–830. doi:10.2166/ws.2010.346.
Stampini, M., A. Salami, and C. Sullivan. 2012. The Water and Sanitation Index of Development Effectiveness (WIDE) in Sub-Saharan Africa: Overview of Country Performance. African Development Bank Africa Economic Brief 3:2, 8pp., http://www.afdb.org/fileadmin/uploads/afdb/Documents/Publications/AEB%20VOL%203%20Issue%202%20FeB%202012_AEB%20VOL%203%20Issue%202%20FeB%202012.pdf.
van der Steen, P. 2011. Application of Sustainability Indicators within the framework of Strategic Planning for Integrated Urban Water Management. Edited by SWITCH. Delft, the Netherlands: UNESCO-IHE Institute for Water Education, http://www.switchurbanwater.eu/outputs/pdfs/W1-1_CALE_MAN_D1.1.7_Indicators_Manual.pdf.

Stoeglehner, G., P. Edwards, P. Daniels, and M. Narodoslawsky. 2011. The water supply footprint (WSF): a strategic planning tool for sustainable regional and local water supplies. *Journal of Cleaner Production* 19: 1677–1686. doi:10.1016/j.jclepro.2011.05.020.

Storer, T., G. White, L. Galvin, K. O'Neill, E. van Looij, and A. Kitsios. 2011. *The Framework for the Assessment of River and Wetland Health (FARWH) for flowing rivers of south-west Western Australia: project summary and results, Final report. Water Science Technical Series*. Western Australia: Department of Water, https://water.wa.gov.au/__data/assets/pdf_file/0008/3896/100214-FARWH2.pdf.

Sullivan, C., and J. Meigh. 2005. Targeting attention on local vulnerabilities using an integrated index approach: the example of the Climate Vulnerability Index. *Water Science and Technology* 51: 69–78.

SWRR (Sustainable Water Resources Roundtable). 2008. *Statistical Compendium to Populate the SWRR Indicator Framework*, http://acwi.gov/swrr/Rpt_Pubs/SWRR-Indicators-Feb05Draft-Part1and2combined_new.pdf.

TetraTech. 2010. *Water Supply Sustainability Index*. NRDC (Natural Resources Defense Council), http://rd.tetratech.com/climatechange/projects/doc/Tetra_Tech_Climate_Report_2010_lowres.pdf.

Tipa, G., and L. Teirney. 2006. *A Cultural Health Index for Streams and Waterways: A tool for nationwide use*. Wellington, New Zealand: Ministry for the Environment, New Zealand, https://www.mfe.govt.nz/sites/default/files/cultural-health-index-for-streams-and-waterways-tech-report-apr06.pdf.

UNEP, and UNEP-DHI. 2015. *Transboundary River Basins: Status and Future Trends*. Nairobi: UNEP, http://twap-rivers.org/#publications.

UNESCO-IHP (UNESCO International Hydrological Programme), IGRAC (International Groundwater Resources Assessment Centre), and WWAP (World Water Assessment Programme). 2012. *GEF Transboundary Waters Assessment Programme (TWAP) Methodology and Execution Arrangements: Transboundary Aquifers and SIDS Groundwater Systems*, http://isarm.org/files/twap-methodology-groundwater-component-revised-aug-2012pdf.

Veolia. 2009. *Water Impact Index (WiiX), http://gb.waterimpactindex.com/blobs/com.cardiweb.cardiboxv6.cm.business.Article/2138115574098558996/doc/1/en/About%20the%20WIIX%20Tool_1.pdf*.

Vörösmarty, C. J., P. B. McIntyre, M. O. Gessner, D. Dudgeon, A. Prusevich, P. Green, S. Glidden, S. E. Bunn, et al. 2010. Global threats to human water security and river biodiversity. *Nature* 467: 555–561. doi:10.1038/nature09440.

Vrba, J., and A. Lipponen. 2007. *Groundwater resources sustainability indicators. IHP-VI Series on Groundwater*. Paris, France: UNESCO, http://unesdoc.unesco.org/images/0014/001497/149754e.pdf.

Wada, Y., and M. F. P. Bierkens. 2014. Sustainability of global water use: past reconstruction and future projections. *Environmental Research Letters* 9: 104003. doi:10.1088/1748-9326/9/10/104003.

Walmsley, J. J. 2002. Framework for measuring sustainable development in catchment systems. *Environmental Management* 29: 195–206. doi:10.1007/s00267-001-0020-4.

WBCSD (World Business Council for Sustainable Development). 2015. *Global Water Tool*, accessed on July 30, 2015, http://www.wbcsd.org/work-program/sector-projects/water/global-water-tool.aspx.

Williams, M., B. Longstaff, C. Buchanan, R. Llanso, and W. Dennison. 2009. Development and evaluation of a spatially-explicit index of Chesapeake Bay health. *Marine Pollution Bulletin*

59: 14–25. doi:10.1016/j.marpolbul.2008.11.018.
WWAP (United Nations World Water Assessment Programme). 2015. *The United Nations World Water Development Report 2015: Water for a Sustainable World*. Paris: UNESCO, http://www.unesco.org/new/en/natural-sciences/environment/water/wwap/wwdr/2015-water-for-a-sustainable-world/. doi:978-92-3-100098-0.
WWF Canada. 2013. Freshwater Health Assessment: Taking the Pulse of our Living Waters, http://awsassets.wwf.ca/downloads/factsheet_fha_final_090913.pdf.
Xu, F. L., Z. Y. Zhao, W. Zhan, S. S. Zhao, R. W. Dawson, and S. Tao. 2005. An ecosystem health index methodology (EHIM) for lake ecosystem health assessment. *Ecological Modelling* 188: 327–339. doi:10.1016/j.ecolmodel.2005.01.058.

Table S2. Indices' component sub-categories, numbers, weighting methods, and geographic scale.

Project/Index name	Components or sub-categories of index	Number of indicators	Weighting method applied	Geographic scale of application
Adaptive Capacity Index	Natural capacity; Physical capacity; Human capacity; Economic capacity	7	Expert opinion	Country
Agricultural Water Poverty Index	Resources; Access; Capacity	16	Equal	Farm
Aqueduct Water Risk Atlas	Quantity; Quality; Regulatory and Reputational Risk	12	Expert opinion	Global
Arab Water Sustainability Index	--	8	Statistical	Country/Regional
Arctic Water Resource Vulnerability Index	Physical; Social	9	Equal	Region
Asian Water Development Outlook	Household; Economic; Urban; Environmental; Resilience to Disasters	17	Population-weighted	Country
Assessing Ecosystem Services in the Goulburn Broken Catchment	Ecosystem Services; Social and Cultural; Economic	15	--	Basin
Bay Health Index	Water Quality; Biology	6	Equal	Estuary
Benthic Index of Biotic Integrity	Community structure and composition; Processes	13	Equal	Basin
Blue Water Sustainability Index	Consumptive blue water use; Nonrenewable groundwater abstraction; Surface water over-abstraction	7	--	Global
California Water Sustainability Indicators Framework	Water supply reliability; Water quality; Ecosystem health; Adaptive & sustainable management; Social benefits & equity	120	--	Sub-national (state)
Canada Water Sustainability Index (C-WSI)	Resource; Ecosystem Health; Infrastructure; Human health; Capacity	15	Equal	Community
City Blueprints	Water security; Water quality; Drinking water; Sanitation; Infrastructure; Climate robustness; Biodiversity/attractiveness; Governance	24	--	City
Climate Vulnerability Index (CVI)	Resource; Access; Capacity; Use; Environment; Geospatial	21	User-defined	Community
Corporate Responsibility Performance Indicators	Company, business principles, policy; Quality & customer service; Resource protection & economy; Employees; Social responsibility; Economic efficiency	30	--	Sector
Cultural Health Index for Streams and Waterways	Site status; Mahinga kai; Cultural stream health	13	Equal	Basin
Cumulative threat indices	Incident human water security threat; Incident biodiversity threat; Investment benefits factor	28	Expert opinion	Global
Ecosystem Health Index Methodology	Phytoplankton; Zooplankton; Exergy	5	Relation-weighting index	Lake
Enhanced Water Poverty Index (eWPI)	Resources; Acces; Use; Capacity; Environment	13	Equal	Basin
EU Water Framework Directive Indicators	Ecological quality; eutrophication and organic pollution; hazardous substances; water quantity	57	--	Country/Regional
Fish-based index for the assessment of river health	Species richness; Faunal composition	11	Equal	Country
Flood Resilience Index (FRI)	Proaction; Preparation; Response; Recovery	24	PCA	City

Framework for Assessing River and Wetland Health	Catchment Disturbance; Hydrological Change; Water Quality; Physical Form; Fringing Zone; Aquatic Biota	21	Equal or Euclidean distance	Country
Framework for Measuring Sustainable Development in Catchment Systems	Driving forces; Pressures; State; Impact; Response	32	--	Country
Framework for Sustainability Analysis in WRM	Risk; Environment; Equity; Economic acceptability	7	Equal	Basin
Framework of Sustainable Water Resource Management Indicators	Water resources; Management; Economic activities & disaster prevention; Institutional & legal arrangement	35	Expert opinion	Basin
Fraser Basin Sustainability Indicators	Aboriginal & non-Aboriginal relations; Agriculture & Food; Air Quality; Biodiversity; Business & Sustainability; Climate Change; Community Engagement; Consumption & Waste; Economy; Education; Energy; Fisheries; Water Quality & Quantity; Population; Income & Employment; Housing; Health; Forests & Forestry	54	--	Basin
Freshwater Health Assessment (FHA)	Water quality; Water flow; Fish; Benthic invertebrates	4	Equal	Country (Canada)
Freshwater Provisioning Index for Humans	Surface water volume; Population	2	--	Global
Groundwater Resources Sustainability Indicators	--	10	--	Various
H2Oe	--	2	--	Product/Process
Human Impact on Freshwater Ecosystem Services	Biodiversity; Commodities; Disturbance regulation; Gas regulation; Water Quality; Water Supply	8	Literature	Global
Index of Biotic Integrity	Species composition and richness; Ecological considerations	12	Equal	Watershed
Indicators for assessment of Integrated Lake Basin Management	Policy; Institutions; Participation; Technology; Information; Finances	42	--	Basin
Indicators of Hydrologic Alteration	Magnitude of monthly water conditions; Magnitude and duration of annual extreme water conditions; Timing of annual extreme water conditions; Frequency and duration of high and low pulses; Rate and frequency of water condition changes	64	--	Ecosystem
Life cycle assessment for urban water systems	Freshwater withdrawal; Production/distribution/use of drinking water; Collection & treatment of wastewater; Handling of bioproducts	15	--	City
MCE of Water Resources Sustainability	Economic efficiency; Social equity; Environmental conservation; Maintenance capacity	16	Expert opinion	Basin
Measuring sustainability in Israel's water system	Hydrologic sensitivity; Management vulnerability; Societial susceptibility	3	--	Country (Israel)
Multi-regional input-ouput model	Carbon; Ecological; Water	3	--	Global
Multiple taxonomic groups to index ecological condition of lakes	Diatoms; Benthos; Birds; Diatoms; Fish; Zooplankton	29	Equal	Lake
National Water Quality Index	Dissolved oxygen; pH; Conductivity; Nitrogen; Phosphorous	10	Equal	Global
Performance Index for Assessing Urban Water Systems	Effective exploitation; Environmental carrying capacity; Meeting consumer demand; Environmental impact	7	Fuzzy inference	City

Performance Indicators for Irrigation and Drainage	Water balance; Environmental sustainability & drainage; Maintenance; Socio-economic performance	40	--	Various
Performance of RBOs	Coordinated decision-making; Responsive decision-making; Financial sustainability; Organizational design; Training & capacity building; Information; Private/public sector roles	24	AHP	Basin
Planktonic Index of Biotic Integrity	Season; Salinity	12	Equal	Estuary
Post-2015 WASH indicators	Open defecation; 2030 targets for drinking water and sanitation facilities; 2040 targets for facilities; social/fiscal/environmental sustainability of services	14	--	Country
Rapid Appraisal Wetland Condition Index	Soil; Fringing vegetation; Aquatic vegetation; Water quality	13	Equal	Wetland
RBO Performance Indicators	Coordinated decision-making; Responsive decision-making; Financial sustainability; Organizational design; Goals & goal shift; Role of law; Training & capacity building; Information & research; accountability & monitoring; Private and public sector roles	115	--	Basin
River Basin Health Indicators (RHA)	Ecology; Water Quality; Physical form; Socioeconomic factors	27	Expert opinion	Country (China)
River Basin Sustainability Index	Social development; Environmental; Biodiversity	6	Expert opinion	Basin
Rural water supply sustainability indicators	Demand responsiveness; Project-related factors; External factors; Sustainability	15	Expert opinion	Community
Spatial Distribution of Water Resource Vulnerability in the Columbia River Basin	Supply; Demand; Quality	21	Equal	Basin
Spatial Water Resources Vulnerability Index	Flood damage; Drought damage; Water quality deterioration	15	Expert opinion	Basin
Spatially Distributed Water Stress Index	--	3	--	Country
Stream-Wetland-Riparian Index	Floodplain-wetland condition; Stream	7	Equal	Basin
Sustainability Assessment Tool for community-managed rural water systems	--	8	--	Community
Sustainability Index	--	3	User-defined	Not specified
Sustainability Index for Integrated Urban Water Management	Social; Economic; Environmental; Institutional	16	User-defined	Community
Sustainability Index for Water Resources Planning and Management	--	4	User-defined	Basin
Sustainability Index of WASH Interventions	Institutional; Management; Financial; Technical	12	Expert opinion	Country and district
Sustainability Indicators for a small lake basin in Western Mexico	Urban development; Public health; Poverty; Education; Industry; Agriculture	52	--	Lake
Sustainability Indicators for WRM in Brazil	--	18	--	Basin
Sustainability Wheel	Regional development; Ecological integrity; Justice; Adaptive capacity	16	--	Basin
Sustainable Rivers Audit	Fish; Macroinvertebrates; Vegetation; Physical Form; Hydrology	14	Expert opinion	Basin

SWITCH Indicators for Urban WM	External; Pressure; State; Response	68	--	City
SWRR Indicator Framework	Water availability; Water quality; Human uses and health; Environmental health; Infrastructure and institutions	14	--	Country (U.S.)
Tiered Framework for Assessing Groundwater Ecosystem Health	Functional; Organizational; Stressors	8	Equal	Aquifer
TWAP Groundwater	Constraints; Importance; Changes; Drivers & Pressures; Enabling Environment; Implementation	20	Equal	Basin
TWAP Lakes	Biophysical conditions; Human use; Institutions; Policies; Participation; Technology; Information; Finance; Planning	19	Equal	Basin
TWAP River Basins	Quantity; Quality; Ecosystems; Governance; Socio-economic	15	Equal	Basin
UN World Water Development Report	Demographics; State of freshwater resources; Water demand; State of the environment; Human well-being; Electricity; Impact of hazards; Progress toward MDGs	32	--	Country
Urban Water System Sustainability	Social; Environment; Economic; Governance; Infrastructure	35	User-defined	City
Vulnerability Index (VI)	Variation; Scarcity; Exploitation; Pollution; Natural Capacity; Physical Capacity; Human Resources Capacity; Economic Capacity	11	Expert opinion and data driven	Basin
Water and Sanitation Index of Development Effectiveness	Resources; Progress	8	Equal	Country
Water and Sanitation Sustainability Index	Place; Permanence; Persons	9	--	Sector
Water Footprint	Green water; Grey water; Blue water	6	--	Various
Water Footprints of Cities	Virtual water imports; Amounts; Import distance	3	--	City
Water Impact Index	Volume; Scarcity; Quality	3	Equal	Industry
Water Impact Index (WiiX)	Volume; Stress; Quality	8	--	Community
Water Management Sustainability Indicators	Environmental; Economic; Social	9	--	Basin
Water Management Towards Sustainability	Economic; Ecological; Social	14	--	Country
Water Poverty Index (WPI)	Resources; Acces; Use; Capacity; Environment	22	User-defined	Community
Water Resources Management Sustainability Index Tool	Governance; Financial; Technical; Environmental; Socioeconomic	21	Equal	Country
Water Risk Filter	Scarcity; Pollution; Impact on ecosystem; Dependence on hydropower; Supplier risk; Regulatory; Reputational	48	Industry-specific	Corporate portfolio
Water Scarcity and Allocation Indices	Economics; Supply; Demand	13	Expert opinion	Basin
Water Scarcity Index	--	3	--	Basin
Water Stress Index	Human health; Ecosystem quality; Resources	5	Eco-indicator 99 default weights	Basin
Water Supply Footprint	--	7	--	Basin

Water Supply Sustainability Index (WSSI)	Water demand; Groundwater use; Susceptibility to drought; Freshwater withdrawals; Summer water deficit	5	Equal	County (U.S.)
Water Sustainability Indicators for Abu Dhabi	Water availability; Water quality; Water use efficiency; Policy & governance	19	--	Sub-national (emirate)
Water Use Benefit Index	Physio-chemical; Social & economic; Ecological	16	User-defined	Basin
Water Vulnerability Index (WVI)	Resource; Extreme event; Land cover; Storage; Demographic; Household; Economic; Bulk demand	16	Expert opinion	Basin
Watershed Sustainability Index (WSI)	Hydrology; Environment; Life; Policy	24	User-defined	Basin
WBSCD Global Water Tool (GWT)	--	15	--	Global
West Java Water Sustainability Index	Conservation; Water Use; Policy & Governance	13	User-defined	Province
